# Selection of Patients with Tourette Syndrome for Deep Brain Stimulation Surgery

**DOI:** 10.3233/BEN-120288

**Published:** 2013

**Authors:** Mauro Porta, Andrea E. Cavanna, Edvin Zekaj, Francesca D'Adda, Domenico Servello

**Affiliations:** ^1^Tourette CentreIRCCS Galeazzi HospitalMilanItaly; ^2^The Michael Trimble Neuropsychiatry Research GroupDepartment of NeuropsychiatryBSMHFT and University of BirminghamBirminghamUK; ^3^Sobell Department of Motor Neuroscience and Movement DisordersUCL and Institute of NeurologyLondonUK; ^4^Functional Neurosurgery UnitIRCCS Galeazzi HospitalMilanItaly

**Keywords:** Deep brain stimulation, Tourette syndrome, tics, co-morbidities, selection criteria

## Abstract

Deep brain stimulation (DBS) is an emerging therapeutic option for severe resistant Tourette syndrome (TS). To date, about 100 cases have been reported in the scientific literature. Different clinical guidelines have been proposed for this procedure from both USA and European centres. A number of issues remain unresolved, mainly in relation to eligibility criteria of patients with TS. We highlight the need for a comprehensive assessment of associated co-morbidities, which are not considered integral part of the syndrome and are not sufficiently evaluated in relation to DBS. The concept of refractoriness, the minimum age of candidates, and the optimal targets for DBS are also controversial.

## 1. Introduction

Tourette syndrome (TS) is a chronic neurodevelopmental disorder defined by the presence of multiple motor tics and at least one phonic tic [1]. It is estimated that about 90% of patients have at least one co-morbid behavioural symptom, such as attention deficit hyperactivity disorder (ADHD), obsessive-compulsive disorder (OCD), self-injurious behaviour (SIB), impulse dyscontrol and affective dysregulation [2]. Overall, the course of tic symptoms is relatively favourable over time: up to 50% of patients with onset of tic disorder before age 10 experience a significant decrease in tic severity during adolescence [3]. However, severe TS can have devastating effects on perceived well being and relationships. Behavioural strategies, in addition to both standard and innovative pharmacological therapies, are the main treatment interventions for patients who continue to experience severe symptoms with significant social impairment.

When patients are refractory to conservative treatments, deep brain stimulation (DBS) could be considered as an emerging therapeutic option. Over the last few decades, DBS has been demonstrated to be a safe and efficacious procedure in different movement disorders, including Parkinson disease, essential tremor and dystonia [4]. More recently, DBS has also been used to treat patients with severe and refractory psychiatric disorders, such as OCD and depression [5].

The rationale for choosing specific targets for DBS to treat patients with severe and refractory TS was initially based on the effects of historical procedures of ablative surgery, which are no longer ethically acceptable [6]. The results of preliminary reports on DBS in TS are encouraging, even though many aspects have yet to be better clarified, in primis the issue of patient selection [7].

## 2. Assessment

Candidates to DBS in TS should undergo a comprehensive neurological, neuropsychiatric and neuropsy-

ISSN 0953-4180/13/$27.50 © 2013 – IOS Press and the authors. All rights reserved chological pre-surgical assessment, to be carried out by a multidisciplinary team with clinical experience in TS and in collaboration with functional neurosurgeons. Detailed clinical assessment guidelines of tic disorders and common co-morbidities are discussed in the recently published guidelines from the European Society for the Study of Tourette Syndrome (ESSTS) [8]. Patients should be diagnosed according to DSM IV-TR criteria and the diagnosis should be validated by instruments such as the TS Diagnostic Confidence Index. With regards to the clinical picture, the tic symptoms should represent the primary problem significantly affecting Quality of Life (QoL).

Tic severity should be evaluated by means of direct observation (e.g. videorecording) and clinical scales, such as the Yale Global Tic Severity Scale (YGTSS). A YGTSS total tic severity score above 35/50 during repeated assessments for at least six months is a reliable indicator of a degree of tic severity prompting consideration of DBS.

The large majority of patients with TS have comorbid psychopathology, including attention-deficit and hyperactivity disorder (ADHD), obsessive-compulsive disorders (OCD), anger control problems, mood disorders, conduct and oppositional defiant disorders. Co-morbidities have to be evaluated using specific scales: for example, the Yale-Brown Obsessive Compulsive Scale (YBOCS) has often been used for evaluation of obsessive compulsive behaviours; the State-Trait Anxiety Inventory (STAI) assesses patients' state and trait anxiety, and the Beck Depression Inventory (BDI) reliably measures depression. It should always be considered that co-morbidity can represent the main reason to seek help in patients with TS. The disease-specific health-related quality of life scale for TS (GTS-QOL) developed by Cavanna et al. [9] highlights the relevance of the patient's subjective point of view. This scale comprises 27 items which examine four components of quality of life: the psychological domain, the physical/activities of daily living (ADL) domain, the obsessive-compulsive domain and the cognitive domain. Importantly, this scale can adequately and specifically evaluate aspects of social impairment which often motivate DBS in refractory patients. The GTS-QOL also captures other aspects of the TS-related impact on life. Self injurious behaviour (SIB) is frequently present in the context of TS. For example, SIB of different severity was reported in 46.1% of patients from a clinical sample of 639 subjects [10]. Patients with life-threatening tics are classified as having "malignant TS". Jankovic et al. [11] in their experience reported that 5% of patients with TS can present malignant TS, often in association with severe obsessive-compulsive symptomatology. Finally, the GTS-QOL can prove useful both as baseline and outcome measure, also in the longer term. For instance, a recent study showed that young patients with severe tics associated with characteristic premonitory urges and a family history of tics disorders appear to be at higher risk for poorer quality of life as adults [12].

Both environmental and psychosocial stressors have to be evaluated as factors aggravating clinical symptoms. Surgical candidates should experience tics associated with a significant functional impairment.

"TS by history" has to be carefully evaluated by asking patients, parents and caregivers. These data can be highly informative, although the value of these parameters is not yet formally validated. At least familiar, environmental and economic issues have to be carefully examined as they are important factors that may influence patient compliance. Analysis of the phenomenological aspects of TS according to Jankovic [13] seems essential to choose targets for DBS; videotaping according to Kompoliti and Goetz [14] and accelerometry [15] can be used to objectively evaluate tic symptoms. The patient or his/her legal representative must given written informed consent before the DBS procedure. If DBS is performed in the context of a trial, the study design has to be approved by the local ethics committee [16].

## 3. Treatment refractoriness

The exact definition of treatment refractoriness in TS has been debated [17]. A significant percentage of patients with TS show spontaneous improvement up to a complete disappearance of most if not all the symptoms during adulthood: DBS is of course indicated for those patients failing to show such improvement of symptoms and related social impairment [18]. The therapeutic strategies used to treat tics and co-morbidities are far from being standardized; although there are many open studies on pharmacotherapy, only a limited number of studies meets rigorous criteria for therapeutic trials. Cochrane reviews and expert views on the pharmacological treatment of TS have recently been published (Fig. 1) [19,20].

Drug therapy often involves the use of antipsychotic agents, which can be associated with a higher risk of adverse effects compared to other drugs. Side effects may persist during adulthood: this fact by itself represent an important element in evaluating refractori-

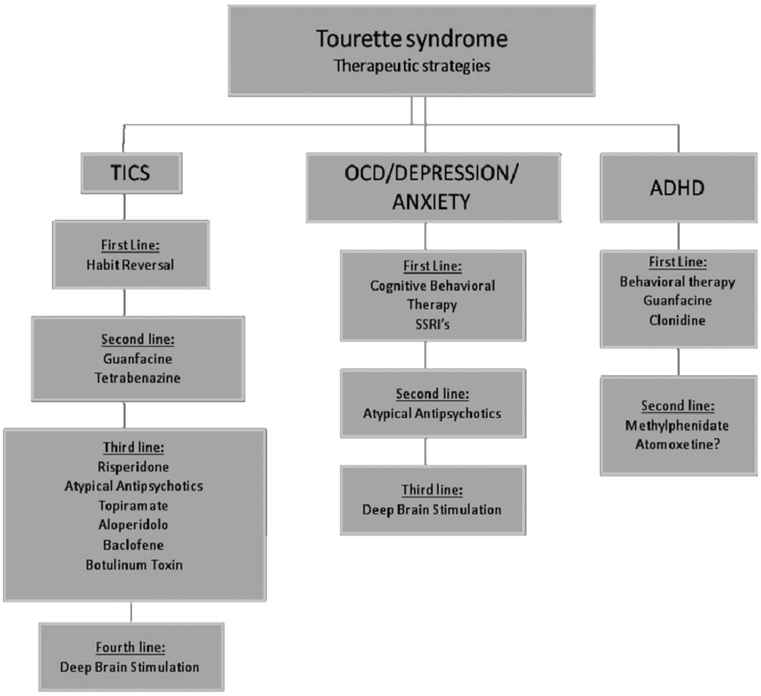

Fig. 1. Proposed algorithm for the treatment of Tourette syndrome, based on the authors' interpretation of evidence-based literature and their own experience (cfr. [20]).

ness. In the scientific literature, drug protocols used before DBS in patients with TS are rarely reported in details [21,22]. Some authors described the use of "maximum dose of established treatments" [23], or "an inadequate response to at least two dopamine blockers or catecholamine depletors" [24], or "failure of best treatment by medication (antipsychotics), or intolerance after a minimum of 6 months of treatment", before DBS [25]. In our department (IRCCS Galeazzi, Milan, Italy), patients with TS are considered treatment-refractory when they failed to respond to a behavioural intervention, such as habit reversal training, and therapeutic doses of at least three medications of proven efficacy for tics, including antidopaminergic drugs (i.e. haloperidol, pimozide, risperidone, tiapride, sulpiride, aripiprazole), $\alpha$-2 adrenergic agonists (i.e. clonidine and guanfacine), and SSRIs (selective serotonin reuptake inhibitors). Refractoriness is also established by lack of response to innovative drugs, which are often prescribed off label.

## 4. Patient age

Age is an issue of particular relevance for the selection of surgical candidates in TS, since spontaneous remission by the age of 18 has been shown to occur in nearly half of the patients [3]. Performing DBS surgery too early in a patient who can undergo spontaneous improvement means to expose him/her to unnecessary surgical risks, despite the fact that DBS is a mini-invasive, reversible, adjustable procedure. On the other hand, delaying the intervention in patients with severe TS symptoms can expose them to a considerable disadvantage, in terms of quality of life, education, and social inclusion. It is well known that the age from 18 to 25 years is a crucial phase for schooling and vocational qualification, thus the presence of severe tics and comorbidities can lead to far-reaching consequences, including permanent social impairment [26,27]. In fact, it is during developmental age that clinical stigmata of the disorder cause the most significant damage, severely and permanently altering the social functioning of the patient, in some cases to such an extent that even after regression of symptoms return to normal social life is almost impossible.

## 5. Target choice

At least eight targets for DBS in patients with TS have been reported. Most studies report experience with small case series (less than five). Overall, in this decade DBS for TS has brought more questions than answers. In our department, fifty patients with TS have been treated with DBS. The most targeted area was the ventro-oralis-centromedian-parafascicular nucleus of the thalamus Vo-Cm-Pf (Lat: 5 mm, AP: −2 mm, Z: 0 mm), with optimal results concerning tics and improvement in OCD and anxiety [28,29]. Stimulation of the nucleus accumbens-anterior limb of the internal capsule (NA-ALIC) was used as first line procedure or as rescue procedure for patients with relevant OCD who failed to respond to Vo-Cm-Pf stimulation. In selected patients with prevalence of dystonic tics, significant reduction of tic severity was obtained targeting the posterior portion of the globus pallidus-pars interna (Gpi). In patients with a clinical picture characterised by significant co-morbidities, we targeted the anterior portion of the GPi [30]. In our opinion, targets should be selected case by case on the basis of the most relevant symptoms (tailoring approach), as different targets seem to have different clinical effects.

## 6. Compliance

Poor patient and caregivers compliance should be considered as a major exclusion criterion. Patient with history of drug addiction have to be carefully evaluated. The role of caregivers in determining outcome of DBS is poorly elucidated. Frequently caregivers may not appreciate amelioration of symptomatology after DBS. The risk is to produce frustration on the patients and decrease the compliance. In most cases physicians focus on patient compliance, while caregiver's compliance is frequently underestimated. From our experience we have learnt that caregiver compliance can be as important as patient compliance. We have had two disposal removals due to caregiver's poor compliance which prevailed over patient will, convincing them of the failure of the surgical procedure. In order to be eligible for DBS, patients should demonstrate compliance with previous treatments [31]. Compliance should be assessed in terms of adherence to pharmacological protocols and psychobehavioural training programs, and attendance to follow-up visits.

## 7. Other exclusion criteria

A controversial issue within exclusion criteria is the finding of brain abnormalities on pre-operative MRI scans. In clinical series of dystonic patients, focal signal abnormalities in the basal ganglia do not contraindicate DBS [32]. On the other hand, in Parkinson disease the presence of chronic ischemic disease or diffuse cerebral atrophy compatible with atypical Parkinsonism represent exclusion criteria for DBS. In our TS series, the presence of vascular abnormalities affected the positioning of the stimulating electrode near the target [33]. There are uncertainties also with regards to general medical conditions representing contraindication to DSB. In various reports, cardiovascular, pulmonary or hematological disorders are considered a contraindication for surgery. In our opinion, patients should be excluded from surgery if they have an American Society of Anesthesiologists (ASA)score of 3 or above which represent an increased risk for general anaesthesia.

Another important aspect related to exclusion criteria is the presence of severe psychiatric co-morbidity. With regards to this, it has to be considered that about 90% of patients with TS have at least one co-morbid behavioural symptom, such as ADHD, OCD, SIB, impulse dyscontrol and affective dysregulation [2]. The scientific literature is controversial about psychiatric disorders representing exclusion criteria for DBS. For instance, Mink et al. [16] recommend that candidates to DBS must not have co-morbid psychiatric disorders, while Shahed et al. [30] proposed that psychiatric comorbidities in TS should not necessarily represent an exclusion criterion, because it is possible that these behavioural problems will also improve with DBS. It is mandatory to evaluate psychiatric disorder case by case, while previous suicidal attempts should be considered a contraindication.

## 8. Conclusion

Different guidelines on the selection of candidates for DBS in TS have been published to date (Table 1).

From the critical evaluation of these guidelines emerges a substantial difference regarding the minimum age at which DBS might be considered. As stated above, we believe that surgical procedure have to be proposed to younger patients, starting from the age of 18 years. It should also be highlighted that exceptions in terms of younger patients may be considered for DBS when needed, as in the case of the 16-year-old patient reported by Shahed et al. [30]. The second problem concerns the behavioural spectrum of TS, which is referred to as co-morbidity. The phenotype of TS

Table 1

Published guidelines for the selection of candidates for deep brain stimulation in Tourette syndrome

| | TSA-American Guidelines [18] | Dutch-Flemish Guidelines [34] | Italian Guidelines [35] | European Guidelines [16] | UK perspective [36] |
|---|---|---|---|---|---|
| Year of publication | 2006 | 2006 | 2009 | 2011 | 2011 |
| Patient's age | 25+ rare exceptions | 25+ | 18+ rare exceptions | 18+ | 20+ |
| Diagnosis | No severe adult cases that were mild-moderate in childhood | Two independent raters using DSM-IV and Diagnostic Confidence Index | Trained rater using DSM-IV-TR | TS or chronic tic disorder diagnosed by an experienced clinician | Multidisciplinary team – DSM-IV-TR<br>Diagnostic Confidence Index |
| Symptoms severity | YGTSS>35/50 for 12 months, videotaping and family interview | YGTSS>20/50 and Social Impairment>5/50, videotaping | YGTSS$\geqslant$35/50<br>Motor tic subscore$\geqslant$ 15, tics causing distress, SIB | YGTSS$\geqslant$35/50, tics should be severe for at least 1 year and lasting at minimum 5 years before DBS | YGTSS<br>$\geqslant$35/50, at least 12 months for surgery. Little hope for spontaneous improvement. Repeated evaluations of QoL |
| Co-morbidities | Baseline assessment of co-morbidities and stable treatment for 6 months | Tics are primary problem (e.g. not OCD or SIB as primary problem) | Extensive assessment to identify potential complications (e.g. somatisation) | Tics are primary problem, co-morbidities (ADHD, OCD, depression, anxiety, SIB) have been treated according to respective guidelines | Structural brain anomalies and coagulopathies, OCD and ADHD are highly prevalent in TS |
| Treatment refractoriness criteria | Failure to respond to (or severe side effects from): $\alpha$-2 adrenergic agonist, typical and atypical antipsychotics and benzodiazepines, behavioural therapy | No/partial response to 3 medications (for 12 weeks): typical and atypical antipsychotics and 'experimental' drug, 12 sessions of behavioural therapy (Habit Reversal Therapy and Exposure and Response Prevention) | Lack of response or intolerable adverse effects to typical and atypical neuroleptics and innovative treatments | Treatment with 3 different drugs including both typical and atypical neuroleptics in adequate dosage over an adequate period of time does not result in significant tic reduction or leads to unacceptable adverse effects. Behavioural treatment for 12 sessions without effect | Therapeutic doses of at least 3 medications proven efficacious for tics, including anti-dopaminergic drugs (Haloperidol, Pimozide, Risperidone and Aripiprazole) and $\alpha$-2 adrenergic agonists (Clonidine) |
| Other patient specifications | Willingness to engage in ongoing treatment, good compliance, consider psychosocial difficulties | Ability to make a 'complete decision' to participate, emphasis on ethics and right to withdraw | Assessment of treatment compliance, symptom related difficulties in social life | Tics cause social impairment in QoL (relationships, home environment, school/work). Compliance and stable psychosocial environment | Patients should be receiving stable and optimized treatment for co-morbid conditions for 6 months prior to surgery |
| Patient exclusion criteria | Tics attributable to other disorder, severe/contraindicated medical or psychiatric conditions | Tics attributable to other disorders, severe/contraindicated medical or psychiatric conditions or mental deficiency | Cognitive dysfunction, substance abuse, tics due to medication or other tic disorder, previous brain surgery or abnormal imaging findings | No tics, OCD symptoms or SIB that could cause electrode or stimulator damaging. No major depression and/or suicidal thoughts at time of operation. No other conditions increasing risks in anaesthesia or operative procedures | Pregnancy and the presence of medical or psychiatric conditions |

Abbreviations: YGTSS, Yale Global Tic Severity Scale; QoL, Quality of Life; ADHD, attention-deficit hyperactivity disorder; OCD, obsessive-compulsive disorder; SIB, self-injurious behaviour.

Table 2
Proposed inclusion criteria for deep brain stimulation in Tourette syndrome

| Eligible candidates for deep brain stimulation should: |
|---|
| – Be above 18 years of age (with possible exceptions) |
| – Be carefully and completely assessed |
| – Be diagnosed according to DSM-IV-TR criteria and Diagnostic Confidence Index standards |
| – Be evaluated in terms of preoperative clinical algorithm (1 year before DBS) in repeated sessions |
| – Present tics and coexisting disorders as primary problem |
| – Undergo thorough evaluation of coexisting disorders using appropriate scales |
| – Show a good compliance |
| – Be evaluated also in term of Tourette "by history" |
| – Experience a stable period of severe tics (YGTSS of 35/50 or above) for at least 12 months prior to surgery |
| – Be treatment-refractory |
| – Be treated conservatively for at least 12 months |
| – Not present any other medical contraindications |

is multifaceted, with special attention to behavioural problems. The issue of whether TS should still be considered as a unitary nosological entity represents a procedural priority. Thus the optimal dissection of TS in each patient is fundamental to understand which patients might benefit from DBS.

In conclusion, TS can no longer be considered a motor disorder and, most importantly, a unitary condition as previously thought. In consideration of this and the data discussed above, we propose the inclusion criteria for DBS used in our department (Table 2), which can be considered a possible platform to establish large multicenter trials to build evidence for DBS protocols and targets in TS.
